# Factors in the Transition from Legal to Illicit Drug Use in Young Adults from Northern Mexico

**DOI:** 10.17533/udea.iee.v37n3e11

**Published:** 2019-10-23

**Authors:** Mayra Selene Ozuna Esprinosa, Josefina Saraí Candia Arredondo, María Magdalena Alonso Castillo, Karla Selene López García, Francisco Rafael Guzmán Facundo

**Affiliations:** 1 Nurse, Ph.D. Professor, Universidad Autónoma de Nuevo León, México. Email: mayraozuna@gmail.com Universidad Autónoma de Nuevo León Universidad Autónoma de Nuevo León Mexico mayraozuna@gmail.com; 2 Nurse, Ph.D. Professor, Universidad Autónoma de Nuevo León, México. Email: joy.sc_@hotmail.com Universidad Autónoma de Nuevo León Universidad Autónoma de Nuevo León Mexico joy.sc_@hotmail.com; 3 Nurse, Ph.D. Professor, Universidad Autónoma de Nuevo León, México. Email: magdalena_alonso@hotmail.com Universidad Autónoma de Nuevo León Universidad Autónoma de Nuevo León Mexico magdalena_alonso@hotmail.com; 4 Nurse, Ph.D. Professor, Universidad Autónoma de Nuevo León, México. Email: kslg2001@hotmail.com Universidad Autónoma de Nuevo León Universidad Autónoma de Nuevo León Mexico kslg2001@hotmail.com; 5 Nurse, Ph.D. Professor, Universidad Autónoma de Nuevo León, México. Email: pako2001@hotmail.com, Corresponding author. Universidad Autónoma de Nuevo León Universidad Autónoma de Nuevo León Mexico pako2001@hotmail.com

**Keywords:** street drugs, drug users, risk factors, young adult, personality, social support, social stigma, residence characteristics, surveys and questionnaires, case-control studies., drogas ilícitas, usuários de drogas, fatores de risco, adulto jovem, personalidade, estigma social, apoio social, arreglo de vivienda, inquéritos e questionários, estudos de casos e controles, apoio social.

## Abstract

**Objective.:**

This research sought to analyze the predictive effect of personal (personality traits), social (social support and social stigma) and community factors (characteristic of the neighborhood and exposure to consumption) on the transition of drug use in young adults.

**Methods.:**

Case and control study. The cases were 70 individuals from 18 to 34 years of age who had already transitioned into illicit drug use and the controls were 210 legal drug usuers (tobacco or alcohol) in the same age range who had not had the transition. A data file was applied along with seven instruments that measured the transition and consumption variables.

**Results.:**

Marihuana was the illicit drug of highest transition. It was shown that greater personality traits of neuroticism, extraversion, and openness to the experience meant higher probability of drug use transition; while greater personality traits of agreeableness and conscience meant lower probability for the transition. The characteristics of the neighborhood environment and exposure to the opportunity of consumption increase the probability of the drug use transition. Social support and social stigma influenced negatively upon the drug use transition.

**Conclusion.:**

Personality traits, neighborhood characteristics, exposure to drug use, social support, and the social stigma of drug use are factors that intervene in the transition from legal to illicit drug use.

## Resumo

**Objetivo.:**

Analisar o efeito preditivo de fatores pessoais (características de personalidade), sociais (apoio social e estigma social) e comunitários (características do bairro e exposição ao consumo) sobre a transição do consumo de drogas em jovens.

**Métodos.:**

Estudo de casos e controles. Os casos foram 70 pessoas de 18 a 34 anos que haviam transitado ao consumo de drogas ilícitas e os controles foram 210 pessoas da mesma idade consumidores de alguma droga lícita (tabaco ou álcool) que não hão tido transição. Se aplicou uma cédula de dados e sete instrumentos que mediram as variáveis de transição e consumo.

**Resultados.:**

. A droga ilegal de maior transição foi a maconha. Se mostrou que as de maiores características de personalidade de neuroticismo, extroversão e apertura à experiência foi mais alta a probabilidade da transição do consumo de drogas; enquanto que a maiores traços de personalidade amabilidade e consciência foi menor foi a probabilidade para a transição. As características do entorno de bairro e a exposição à oportunidade de consumo incrementam a probabilidade da transição do consumo de drogas. O apoio social e a estigma social influenciaram negativamente na transição do consumo de drogas.

**Conclusão.:**

Os traços de personalidade, as características do bairro, a exposição ao consumo de drogas, o apoio social e a estigma social do consumo de drogas são fatores que intervém na transição do consumo de drogas legais a ilegais.

## Introduction

Illicit drug abuse constitutes an important risk for the health of individuals due to diverse chronic noncommunicable diseases and, hence, contributes significantly to premature mortality and affects the quality of life of people as it is related with accidents, violence, suicides, assaults, and fights.(1) Currently, a general trend exists of considering the predisposition for illicit drug use as the result of the interaction of personal, community, and social factors,(2) but the initial consumption of alcoholic beverages, tobacco, or illicit drugs considers personal decisions and voluntary behaviors, which is why this would be an interesting approach for prevention by nursing professionals. In that sense, some studies found a higher probability of experimenting with illicit drug use when legal drugs have been consumed previously,(3,4) denominating this phenomenon as the transition from one drug to another. However, these transitions between the consumption of legal to illicit drugs has still not been examined sufficiently.

Within the literature on the phenomenon of drug use, the word “transitions” refers to the passage of using one drug to another, for example from alcohol to tobacco; from alcohol to marihuana; from tobacco to cocaine, and from such to amphetamines.(5) Studies identify some personal and community factors that can influence as facilitators in the drug use transition, like exposure to the opportunity, context of the residence such as characteristics of poor quality of the neighborhood, availability of drugs, perception of lack of social support, dropping out from school, child abuse, family history of consumption and delinquency, consumer peers, low control in situations of consumption, and personality of the subjects.(6,7) 

Due to the aforementioned, it is worth delving into the phenomenon of the transition from legal to illicit drug use, as well as into the predictive influence of some personal and community factors that can help to explain this phenomenon, which can constitute an important target of prevention efforts, given that the occurrence of the event over time helps to understand the value of prevention actions. In Mexico, cross-sectional studies have been conducted that explore, among other topics, the initiation in the use of drugs;(6,7) however, enough investigations are not reported that incorporate in their methodology the analysis of diverse factors that can be facilitators or inhibitors in the transition processes of consumption from one to another drug. This situation permits reflecting on the need to address this phenomenon under perspectives of the nursing discipline, which permit identifying more clearly what factors facilitated the transition from legal drug use to illicit drugs in young consumers. The aforementioned permits characterizing populations in greater risk of drug use to carry out preventive actions aimed at strengthening inhibitor factors of drug use. 

Within the nursing discipline, there is the Transition Theory by Meleis,(8) which conceptualizes the influence of factors that can facilitate or inhibit transitions toward behaviors that generate health or disease. The aim of this study was to analyze the predictive effect of personal (personality traits), social (social support and social stigma), and community factors (characteristic of the quality of the neighborhood and exposure to the opportunity of consumption) on the nature of the transition from legal to illicit drug use in young adults in northern Mexico. 

## Methods

The study design was individual base analytic observational of cases and controls; with hypotheses derived from some proposals by Meleis’ Transition Theory:(8) H1 - personality traits increase the risk of the transition of illicit drug use; H2 - the characteristics of the neighborhood environment and exposure to the opportunity of consumption increase the risk of the drug use transition; H3 - social support and social stigma influence upon the drug use transition.

The study population included individuals from 18 to 34 years of age, from the metropolitan area of Nuevo León, Mexico. The sample calculation was carried out through the nQuery Advisor software 7.0, for a logistic regression conditioned with 0.05 significance level, with bilateral alternative hypothesis, for 25% transition proportion, disparity rate ratio of 1.75, principal covariant ratio of 0.20, and 90% power. The final calculated sample size was of 280 subjects. The study considered 25% of the total simple as cases (*n*=70) and the remaining 75% (*n*=210) were controls.

The selection of the 70 cases was conducted among young adults who have transitioned from legal to illicit drug use, the search for and selection of the participants was through the snowball method and which accepted to participate in the study and signed the informed consent. For this study, the group of 210 controls was gathered from the search for young adults who did not use illicit drugs, but consumed some type of legal drug (tobacco or alcohol) at home, taking as reference the place of residence of each case. 

To collect the data, a Personal Data file was used along with the History of Drug Use plus six instruments. The personal factor of personality trait used the Five-Factor Reduced NEO Personality Inventory (NEO FF-I), which measures five personality traits; neuroticism, extraversion, agreeableness, openness to the experience, and conscientiousness.(9) The social factors (social support and social stigma) used the Social Support Questionnaire(10) (MOS- Medical Outcomes Study) and the Stigma Internalization Scale.(11) The community factors (neighborhood characteristics and exposure to consumption) used the Neighborhood Short Form,(12) and the questionnaire on exposure to the opportunity of consumption.(13) The neighborhood characteristics scale measures quality characteristics, like safety, support, and pride. It should be mentioned that for each instrument the final scores were converted into indices taking values from 0 to 100, where a higher score means a greater factor measured. To measure drug use, the study used the Alcohol, Smoking, and Substance Involvement Screening Test (ASSIST).(14)

This study was approved by Research and Ethics Committees at the Institution to which the authors belong. After obtaining the corresponding authorizations, the search was made for cases; if the subject wished to participate, a screening interview was conducted inquiring if their first drug consumed was a legal drug to verify their eligibility for the study. The process began by gathering information, reading and signing the informed consent, and requesting apart in a blank sheet - and with no possibility of relating it to the participant - their home address to take it as reference for the search for the participants in the control group; subsequently they were given the instruments to be answered. Thereafter, data was collected from the participants in the control group, it should be mentioned that this group was comprised from the reference of the places of residence of the participants in the case group. The place of residence was located and, using the clockwise method, continuous homes were visited to search for three controls per case that fulfilled or not the criterion of not being consumers of illicit drugs while being consumers of some legal drug. Once these were identified, information was gathered and their informed consent was read and signed.

The data obtained were processed through the Statistical Package for the Social Sciences (SPSS^®^), version 20.0 for Mac OSX. Descriptive statistics was used to obtain frequencies, proportions, measures of central tendency and of variability to describe the study population and the variables used within the model. The internal consistency of the instruments was determined through Cronbach's Alpha Reliability Coefficient. The Kolmogorov-Smirnov Goodness of Fit test was used with Lilliefors Correction to determine the normality in the distribution of continuous and numerical variables and, based on these results, non-parametric statistics and Logistic Regression Models were used.

## Results

The mean age among the participants was of 21.4 years (SD=3.3); in relation to sex, 77.1% of the young adults were men and 22.9% women in both groups. Regarding academic level, it was found that the young adults with undergraduate degree represented the highest percentage in both groups (52.4% group without transition 52.9% group with transition), followed by high school level (36.2% and 30.0%, respectively). Seventy percent of the young adults from the control group answered that they lived with both parents and only 44.3% from the case group; 78% (95% CI 69%-88%) of the participants who have transitioned to illicit drug use reported having consumed marihuana, with this substance showing the highest consumption, followed by consumption of sedatives (20%, 95% CI 10%-30%) and consumption of amphetamines (11.4%, 95% CI 4%-19%). 

**Figure 1:**
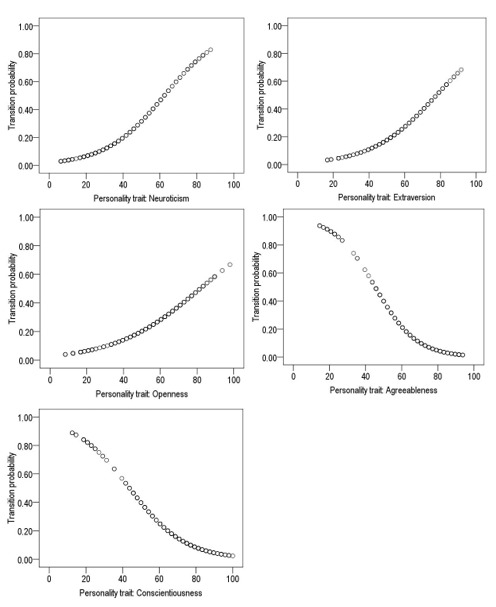
Effect of personality traits on the drug use transition

Table 1 illustrates the Logistic Regression Models between the personality traits and the drug use transition to answer H1. The results show positive effects of the traits of neuroticism, extraversion, and openness to the experience; on the contrary, the personality traits of agreeableness and conscientiousness had negative effects on the transition from legal to illicit drug use. This means that higher personality traits of neuroticism, extraversion, and openness to the experience mean higher probability of the transition of illicit drug use, while a higher personality trait of agreeableness and conscientiousness means lower probability for the drug use transition (Figure 1).

**Table 1:** Binary logistic regression model of personality traits on the drug use transition

							95% CI for	95% CI for
Variables	β	SE	Wald	DF	OR	p-value	OR	OR
							LL	UL
Neuroticism	0.06	0.008	58.69	1	1.06	0.001	1.04	1.08
Constant	-3.91	0.439	79.70					
Model 1	X2=81.33, df=1, R2=37.3%, p<0.001							
Extraversion	0.05	0.010	38.83	1	1.05	0.001	1.03	1.07
Constant	-4.30	0.604	50.84					
Model 2	X2=42.09, df=1, R2=20.7%, p<0.001							
Openness to the experience	0.04	0.008	28.19	1	1.04	0.001	1.02	1.06
Constant	-3.53	0.514	47.05					
Model 3	X2=35.95, df=1, R2=17.8%, p<0.001							
Agreeableness	-0.08	0.011	59.54	1	0.916	0.001	0.89	0.93
Constant	3.96	0.650	37.22					
Model 4	X2=109.53, df=1, R2=47.9%, p<0.001							
Conscientiousness	-0.06	0.009	58.39	1	0.936	0.001	0.92	0.95
Constant	2.90	0.527	30.33					
Model 5	X2=84.42, df=1, R2=38.5%, p<0.001				

Note: β = beta, SE = Standard error, DF = Degrees of freedom, OR = Odds ratio, *p* = Probability, CI = Confidence interval, LL = Lower limit, UL = Upper limit, R2 = Determination coefficient.

**Figure 2:**
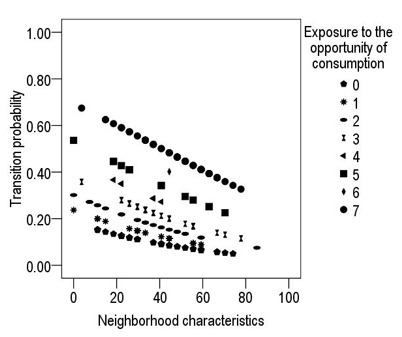
Effect of neighborhood characteristics and exposure opportunity on the drug use transition

According to H2, the results from Table 2 indicate that neighborhood characteristics and exposure to the opportunity of consumption have significant effect on the drug use transition with an explained variance of 17.9%. The results show negative effect of the neighborhood characteristics, while exposure to the opportunity of consumption had a positive effect on the drug use transition, that is, that a lower perception of neighborhood quality means a greater probability of the transition to drug use; however, the probability of the transition increases with higher exposure to opportunities of consumption (Figure 2). 

**Table 2:** Binary logistic regression model of neighborhood characteristics and exposure to the opportunity of consumption on the drug use transition

							95% CI for	95% CI for
Variables	β	SE	Wald	DF	OR	p-value	OR	OR
							LL	UL
Neighborhood characteristics	-0.020	0.009	4.45	1	0.98	0.035	0.96	0.99
Exposure to the opportunity of consumption	0.328	0.059	31.46	1	1.38	0.001	1.23	1.55
Constant	-1.49 0	0.385	15.09					
Model 1	X2=36.07, df=2, R2=17.9%, p<0.001				

Note: β = beta, *SE* = Standard error, *df* = Degrees of freedom, *OR* = Odds ratio, *p* = Probability, IC = Confidence interval, LL = Lower limit, UL = Upper limit, R2 = Determination coefficient.

**Figure 3:**
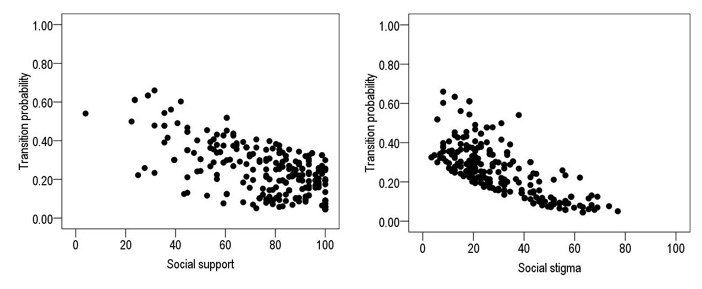
Effect of social support and social stigma on the drug use transition

To answer H3, which proposes that social support and social stigma influence on the transition from legal to illicit drug use, the results are shown in Table 3, finding that social support and social stigma showed significant negative effect on the drug use transition, with an explained variance of 13.2%. These results demonstrate that greater social support and higher perception of stigma on the drug use indicates lower probability for the transition of illicit drug use (Figure 3). 

**Table 3:** Binary logistic regression model del social support and social stigma on the drug use transition

							95% CI for	95% CI for
Variables	β	SE	Wald	DF	OR	p-value	OR	OR
							LL	UL
Social support	-0.023	0.007	11.84	1	0.977	0.001	0.96	0.99
Social stigma	-0.038	0.010	13.89	1	0.963	0.001	0.94	0.98
Constant	1.70	0.615	7.69					
Model 1	X2=26.17, df=2, R2=13.2%, p=0.001			

Note: β = beta, *SE* = Standard error, *df* = Degrees of freedom, *OR* = Odds ratio, *p* = Probability, IC = Confidence interval, LL = Lower limit, UL = Upper limit, R2 = Determination coefficient.

## Discussion

In this study, marihuana was the illicit drug of greater consumption among young adults who have transitioned from legal to illicit drug use. These data agree with official reports in Mexico for 2016 and 2017,(15) highlighting the prevalence of marihuana, which is quite above cocaine, inhalable substances, and hallucinogens. The aforementioned could be explained by the fact that it is the only substance with important growth in recent years, while the rest have had stable behavior. The literature(4-6) shows that marihuana is considered the entry way to the consumption of other illicit substances, denominated “hard drugs”, particularly cocaine and methamphetamines. Besides, an explanation on the high prevalence of marihuana use is that many young adults perceive it as a drug of little or no danger; likewise, in recent years, its legalization has been discussed in diverse public, political, academic, social, and legal settings.(16) 

Regarding H1, which claims that personality traits influence on the transition from legal to illicit drug use, our results support said hypothesis, showing positive effects of the traits of neuroticism, extraversion, and openness to the experience; on the contrary, agreeableness and conscientiousness had negative effects on the drug use transition. As with the results from other studies,(17,18) when the personality traits of neuroticism, extraversion, and openness to the experience are present in an individual’s personality there is a higher probability for illicit drug use, while if the individual has higher personality traits of agreeableness and conscientiousness, the probability can be lower for the drug use transition to occur; this may be due to the harmful influence of lack of scrupulousness in substance abuse behaviors that reveal that lack of control of the behavior, increased impulsivity, and participation in risk behaviors found in the traits of neuroticism and extraversion, characterized by sociability, the need to have many friends, the taste for risk and a tendency to be aggressive, surely through social facilitation processes, and having a high number of friends engaged in behaviors outside the norm are associated with higher consumption during this evolutionary stage. Conversely, the trait of agreeableness is characterized by the capacity for altruism, compassion, and sensitivity with others; these characteristics are to a large measure why they do not show transition and agree with some authors.(19,20) In this sense, it is important for these personality traits to be indicators to bear in mind in the design of prevention programs that promote the development of these traits.

Hypothesis two proposes that the characteristics of the neighborhood environment and exposure to the opportunity of consumption increase the risk of the transition from legal drug use to illicit drug use. The findings permit supporting said hypothesis; according to the neighborhood characteristics, in this study a negative effect was shown, that is, a better perception of the quality in the neighborhood environment indicates lower probability of consumption. These data agree with the literature,(21,22) which indicate that unfavorable neighborhood conditions are a risk factor for the onset of drug use.

With respect to the number of opportunities to use drugs, the highest figures were reported by the young adults who have transitioned to illicit drug use, compared with those who have not transitioned. This is similar to another research(23) which found that exposure to the opportunity of consumption represents a higher risk with the use of drugs. In addition, said study indicates that exposure to the opportunity of consumption can be a cause on the initiation of illicit drug use due to the possibility that this represents of being in front of the substance and the ease of access to it without intentionally looking for it.

Hypothesis three proposes that social support and social stigma influence on the transition from legal to illicit drug use. Results permitted testing the study hypothesis, showing that social conditions, like social support and the perception of social stigma, are factors that inhibit the drug use transition. These results agree with other authors(24,25) that show that when the individual has higher perception of social support and greater perception of the stigma, which is understood as the stereotypes or negative physical, moral, or social prejudice that society implants in young adults who use drugs, there is lower probability to transition from illicit drug use. Social support from the family, peers, and from the environment can function as a protection factor due to the fact that by feeling as part of their environment, can serve to avoid the search for sensations through the drug use. Studies(24,26) have evidenced that inadequate social support in young adults has been related with maladaptive behaviors, including drug use, explaining that social support in function of the different sources of support predict the consumption of different substances. Thus, the results herein point to a protection relationship of social support mainly from the family. Thereby, the family becomes the core of the protection factors of social support against the transition of drug use. That is, the fact of feeling loved, esteemed, and protected by the family members is one of the principal resources the young person has to avoid getting involved or transitioning in the drug use. 

Similarly, higher scores were reported of stigma in the young adults who had not transitioned to illicit drug use. This agrees with diverse studies, which indicate that stigmatization toward consumers of illicit drugs can be an isolation factor, besides they are subject to derogatory stereotypes or criminalization by society.(25,27) This can influence as a protection factor due to the public perception of the stigma in drug consumers, affect social and labor development, and - thus - avoid the rejection an illicit drug user may have. In spite of the results found, which reported that the perception of the stigma diminishes the transition to illicit drug use, studies(25,27) indicate that the stigmatization people who use drugs is marked with negative attributes that damage their social and mental development even when they have stopped consuming and are in rehabilitation, and this brings along a deficit in treatments or places for rehabilitation due to the lack of government support. In Mexico, programs have been developed to avoid discrimination in diverse vulnerable groups, like HIV patients, racial discrimination, among others; however, care for the group of drug users is scarce or null, and drug use is considered a public health problem, but more identified with security than with the individual consumers and their need for care. This may be because drug use is seen as an attitude related with a decision by each individual and not as a disease or ailment.

The prior results permit affirming that proposed in the Transitions Theory by Meleis, which indicates that transition conditions, like personal, community, and social factors or characteristics can facilitate or inhibit the experience of the transition from legal to illicit drug use. Meleis(8) indicates that transitions may be unhealthy and develop over a given time with responsibility to obtain a problematic result against the transition experienced, which will depend directly on the vulnerability presented by the person and on the facilitating or inhibiting conditions of the transition process, such as personal conditions, like beliefs, cultural attitudes, knowledge, and the community and social conditions in which the person is immersed. All of the aforementioned could harm or enhance the transition stage. According to Meleis,(8) these process patterns guide people to health or toward greater vulnerability, this permits nursing to evaluate patterns, like coping strategies to facilitate healthier results.

Nursing professionals are the principal caregivers of clients and families who undergo the transitions from legal to illicit drug use; in this sense, the role of nurses within primary care staff and specialized nurses can develop a leading role in actions regarding promotion of community health and in prevention to avoid the drug use transition in young populations from a multi-factor approach on addictions. Their contribution can be decisive in early detection, the initial approach, referral to specialized resources, and in the coordination and follow up from the different social and health teams, always from continuous communication with the user individual, bearing in mind individual needs and favoring the development of each patient. Nursing professionals, as professionals implied and called to lead these processes, must be capable of integrating the tools of the discipline to solve and avoid the transitions of the drug use.

It is important to recognize that this research has limitations. In the first place, by this being a case and control design, it is not possible to identify direct causality. This urges interpretation of the data with caution because the method was based on retrospective data; concerns are generated on the accuracy of the memory and it is important to consider using other types of designs for future research. Although the use of questionnaires is acceptable to collect information related with the use of substances, it is important to emphasize on the lack of biochemical or physiological indicators, so that the estimation of these variables is according to the answer provided by the participant.

The conclusion here is that the findings contribute to improving the understanding of some factors that intervene in the transition from legal to illicit drug use, such as personality traits, neighborhood characteristics, exposure to drug use, social support, and the social stigma of drug use, which could be useful to develop more effective interventions to prevent drug use. In future studies, it is important to consider a more detailed analysis of the quality and duration of the family relationships because they can be risk factors, as well as protection factors, necessitating their inclusion in future research and, thus, obtain a more complete picture of these relationships during adolescence and youth, to be taken into account in the design of interventions to prevent the transition from legal to illicit drug use. Additionally, we recommend replicating the study with longitudinal designs to broaden the data collection time and have more specific data. This is important because some of these factors are developed during early stages of life when information on these factors could be collected in more detail. 
